# Semen quality of male partners of infertile couples attending a private specialist infertility hospital in Kumasi, Ghana: a retrospective descriptive analysis

**DOI:** 10.11604/pamj.2025.51.54.41953

**Published:** 2025-06-20

**Authors:** John Jude Annan, Mike Addison, Anthony Enimil, Collins Abaitey, Robert Aryee, Augustine Twumasi, Fati Ibrahim

**Affiliations:** 1 Department of Obstetrics and Gynaecology, School of Medicine and Dentistry, Komfo Anokye Teaching Hospital, Kwame Nkrumah University of Science and Technology, Kumasi, Ghana,; 2 Emena Diagnostic and Fertility Center, Aninwah Medical Center, Kumasi, Ghana,; 3 Department of Child Health, School of Medicine and Dentistry, Kwame Nkrumah University of Science and Technology, Kumasi, Ghana,; 4 Department of Statistics, Kwame Nkrumah University of Science and Technology, Kumasi, Ghana

**Keywords:** Semen analysis, male infertility, abnormal semen parameters, male partners, semen quality

## Abstract

**Introduction:**

since the establishment of the fertility center in 2014, no review has been conducted on the pattern of semen abnormalities. This study was therefore aimed at evaluating the range of semen abnormalities in our patient population.

**Methods:**

this was an ethically approved retrospective descriptive study that retrieved and analyzed the semen analysis results of male partners of infertile couples, over a six-year period since the establishment of the fertility center. All data were entered into a Microsoft Excel spreadsheet and transported to R-statistical software version 3.4.2 for statistical analysis. Mean ± standard deviation (SD) was calculated for age, semen pH, volume, concentration, and motility. Categorical variables were represented by frequency (N) and percentages (%). The Chi-square test was used to determine age groups and calendar year´s association with seminal fluid characteristics. Statistical significance was set at p<0.05.

**Results:**

there were 1,224 eligible semen results with a mean age of 39.6 (SD 8.1) and a range of 21-78 years. More than half (56.7%) were very young (less than 40 years). Age group 30-39 had the highest number of clients, 622 (50.8%). The rate of abnormal semen quality was 36%. The most common semen abnormalities were oligospermia 30.5%, teratozoospermia 28.8%, asthenozoospermia 27.3%, hyperviscous semen 18.5%, and hypospermia 20.1%. The mean (SD) of semen volume and sperm concentration differed significantly between the age groups, with a p-value of 0.0000341 for semen volume and a p-value of 0.01 for sperm concentration.

**Conclusion:**

the most common semen abnormalities were oligospermia, teratozoospermia, asthenozoospermia, hyperviscous semen, and hypospermia, with the rate of abnormal semen quality being 36%. More younger men are seeking fertility treatment with their partners.

## Introduction

Infertility is a subject of great importance in a pro-natalist African society. It leads to significant physical, emotional, psychological, and financial morbidity for the couple. These adverse effects also affect the extended family, workplace productivity, and the entire society. Even though advancing knowledge and developments in assisted reproductive techniques have proven that both males and females contribute to the cause of infertility, in the pro-natalist African society, the female partner always remains the first target as the cause of this problem, and she bears the major brunt. There are pervasive societal misconceptions that insinuate that once a man can achieve an erection, sustain it, and ejaculate, then failure to get pregnant connotes a female problem-adequate erection equates to fertility, so the male becomes elusive to investigations and treatment [1]. Therefore, the male usually exhibits some reluctance to lead the female partner to seek fertility care and submit to the only test he has to perform-semen analysis, due to the fear of being branded as infertile, as male infertility leads to a loss of social status and respect in these societies.

The prevalence of infertility in the general population is reported to be 15% - 20%, of which the male factor is responsible for 20% - 40% [2], female factors contribute 30% - 40% while, both factors and unexplained causes account for 20% - 40% each [3].

Male infertility is the inability of the male reproductive cells to produce mature, actively motile, and functional spermatozoa in sufficient amounts that will ensure fertilization of a released ovum in the fallopian tube [4]. The causes of male factor infertility may arise from conditions at the pre-testicular, testicular, and post-testicular levels [5,6]. These conditions usually lead to abnormalities in the semen quantity or quality, leading to abnormalities in semen volume, viscosity, leucocyte count, pH, sperm concentration, count, motility, and morphology. Therefore, seminal fluid analysis may be used as a surrogate measure of male fecundity, and it provides some insight into the underlying pathological problems of male infertility. Therefore, semen analysis has remained an essential, objective, inexpensive, and readily available means of evaluating male factor infertility. This implies that accurate evaluation and treatment of the man is of great importance in addressing this issue of infertility. There are several fertility centers that offer various aspects of infertility treatments in Ghana. However, there is a paucity of studies into the pattern of semen abnormalities at these fertility centers in Ghana. The Emena Diagnostic and Fertility Center at the Aninwah Medical Center, Kumasi, Ghana, commenced specialist fertility services in 2014. Since its inception, the number of patients and couples seeking fertility treatment has been increasing annually, and so is the number of semen analyses performed at this center. However, no review has been conducted to assess the pattern of seminal fluid abnormalities at this center.

This study, therefore, aimed to evaluate the seminal fluid profile of male partners of infertile couples who have been offered fertility treatment at this center, to identify the types of semen quality abnormalities.

## Methods

**Study design and setting:** this was a 6-year retrospective descriptive study on seminal fluid analyses of male partners of women seeking infertility treatment in a private infertility specialist hospital in the Ashanti Region of Ghana between January 2015 and December 2020. The setting of the study was the Emena Diagnostic and Fertility Center. This fertility center was established at The Aninwah Medical Center in 2014. It is located in Kumasi, the second largest city in Ghana, which is the capital of the Ashanti Region of Ghana. The center has clientele from Ghana, the West African subregion, Europe, and North America. The fertility center attends to patients throughout the weekdays (Monday-Friday) and occasionally on weekends. The center is equipped with a modern fertility center and has infrastructure that offers all the various types of modern infertility treatment, including in vitro fertilization, intracytoplasmic sperm injection and gamete donation, surrogacy, and gamete freezing. It also has facilities for minimal access gynaecological surgery for infertility clients.

**Study population:** the scope of the study was limited to male partners of infertile couples who had semen analysis performed at the center during the study period. Included in the analysis were the semen results with a complete dataset based on the study´s objectives, samples that were analyzed based on WHO (2010) criteria (a period of abstinence from sexual intercourse or masturbation for a period of 3 - 5 days, avoidance of the use of antibiotics prior to sample collection, samples collected into sterile screw-capped plastic universal containers and transported to the laboratory within less than 1 hour of production). Excluded from the study were semen results of male partners of women who did not present with infertility, couples with less than 12 months' history of infertility, couples who were not living together or not having regular intercourse, male sperm donors, and record books with inadequate documentation. There was no sample size calculation. All the semen analyses conducted during the study period that fulfilled the inclusion criteria were included in the study.

**Data collection:** the hard and electronic copies of the semen analysis results of male partners of infertile couples who had semen analysis performed at the center during the study period (between January 2015 and December 2020) were retrieved and analyzed. A structured data collection tool was used to collect the data. Variables collected included the age of the patient and macroscopic and microscopic characteristics of the semen. Macroscopic characteristics assessed included semen volume, viscosity, leucocyte count, pH, and appearance or colour. The microscopic characteristics included sperm concentration, sperm count, motility, and morphology. There were no overlaps of male partners in the study period. The data obtained was completely anonymized using code numbers. Coding was done for different years. The data captured as a hard copy was then entered into a Microsoft Excel spreadsheet. The data was cleaned, password-protected, and accessible to only the investigators. No patient-identifiable characteristics were collected.

### Definitions

**Infertility:** the inability of a sexually active, non-contracepting couple to achieve spontaneous pregnancy in one year.

**Male infertility:** the inability of the male reproductive cells to produce mature, actively motile, and functional spermatozoa in sufficient numbers that will ensure fertilization of a released ovum in the fallopian tube.

**Seminal fluid analysis:** a set of descriptive measurements of spermatozoa and seminal fluid parameters that help to estimate semen quality.

**Normospermia:** sperm count of 15 million per milliliter and above.

**Oligospermia:** sperm count of below 15 million per milliliter.

**Azoospermia:** absence of spermatozoa in the ejaculate.

**Asthenozoospermia:** reduced sperm motility of <32%.

**Teratozoospermia:** reduced sperm morphology of <4% normal forms.

**Oligo-astheno-teratozoospermia (OAT):** all variables (count, motility, and morphology) are abnormal.

**Statistical analysis:** all data were entered into a Microsoft Excel spreadsheet and transported to R statistical software version 3.4.2 for statistical analysis. Mean ± standard deviation (SD) was calculated for age groups, sperm pH, volume, concentration, and motility. Categorical variables were represented by frequency (N) and percentages (%). The Chi-square test was used to determine age groups with seminal fluid characteristics. Statistical significance was set at p<0.05.

**Ethical considerations:** ethical approval for the conduct and subsequent publication of the study was obtained from the Institutional Review Board for Research and Development (IRB/R&D) of the Komfo Anokye Teaching Hospital with approval number: KATHIRB/AP/055/21 and the fertility center gave permission for the use of the patient data. The data obtained were completely anonymized using code numbers. The hard copies were securely kept under lock and key, and the electronic data was stored in a password-protected computer. The data were accessible only to the investigators. No patient-identifiable information was documented. There was no envisaged risk to the participants.

## Results

**General characteristics of the study population:** over the period, 1,224 semen analysis results were deemed eligible for inclusion in this study. The mean age (SD) of the study participants was 39.6 (8.1) with a range of 21-78 years. The age group 30-39 years had the highest number of clients, 622 (50.8%). Of the 1,224 participants, more than half, 694 (56.7%), were very young (less than 40 years); 1,077 (88.0%) were less than 50 years old, and 28 (2.3%) were more than 60 years old. This is depicted in Table 1.

**Table 1:** general characteristics of the study population

Age (years)	Number (n)	Percentage (%)
<20	0	0
20 - 29	72	5.9
30 - 39	622	50.8
40 - 49	383	31.3
50 - 59	119	9.7
60 - 69	26	2.1
>70	2	0.2
Total	1224	100
Mean (SD)/years	39.6 (8.1)	
Range	21 - 78	

This table represents the general characteristics of participants: the age distribution and the means (SD) of their ages.

**Seminal fluid characteristics of the study population**: Table 2 shows the seminal fluid characteristics of male partners from 2015-2020. Participants with hyperviscous semen were 227 (18.5%). For sperm concentration, 373 (30.5%) had oligospermia (spermatozoa concentrations less than 15 million per milliliter) while 67 (5.5%) had azoospermia (absence of spermatozoa in the ejaculate). Regarding morphology, 353 (28.8%) were teratozoospermia. Asthenozoospermia was present in 353 (27.3%) of the participants. The most common semen abnormalities were oligospermia 373 (30.5%), teratozoospermia 353 (28.8%), asthenozoospermia 353 (27.3%), hyperviscous semen 227 (18.5%), and hypospermia 246 (20.1%).

**Table 2:** semen fluid characteristics of male partners showing the frequency distribution with 95% confidence intervals of seminal fluid characteristics (macroscopic and microscopic) for the study participants from 2015 - 2020

	n (%)	95%CI
**Viscosity**		
Normal	990(80.9)	78.6-83.1
Hyperviscous	227(18.5)	16.4-20.8
Watery	7(0.6)	0.23-1.2
**Sperm volume**		
Normal	941(76.9)	74.4-79.2
Hyperspermia	37(3.0)	2.1-4.1
Hypospermia	246(20.1)	17.9-22.4
**Sperm concentration**		
Normal	784(64.1)	61.2-66.7
Oligospermia	373(30.5)	27.9-33.1
Azoospermia	67(5.5)	4.3-6.9
Morphology		
Normal	805(65.8)	63.0-68.4
Teratozoospermia	353(28.8)	26.3-31.5
Indeterminate (azoospermia)	66(5.4)	4.2-6.8
**Motility**		
Normal	824(67.3)	64.7-69.9
Asthenozoospermia	334(27.3)	24.8-29.9
Indeterminate (azoospermia)	66(5.4)	4.1-6.8
**PH**		
<7.2	7(0.6)	0.23-1.2
≥7.2	1217(99.4)	98.8-99.8
**Progressive motility**		
<32	443(36.2)	33.3-39.1
≥32	780(63.8)	60.9-66.7

The table shows the frequency distribution with 95% confidence intervals of seminal fluid characteristics (macroscopic and microscopic) for the study participants from 2015 - 2020

**Seminal fluid characteristics compared with age groups:** Table 3 shows the various seminal fluid characteristics compared with age groups for male partners of women seeking infertility treatment. Age groups were compared with semen pH, volume, sperm concentration, and sperm motility. The mean (SD) of volume and sperm concentration differed significantly between the age groups, with a p-value of 0.0000341 for semen volume and a p-value of 0.01 for sperm concentration.

**Table 3:** comparison of semen fluid characteristics between age groups

Age group/parameters	PH mean (SD)	Volume mean (SD)	Sperm concentration (x106) mean (SD)	Sperm motility (PR) mean (SD)
<30	8.18(0.3)	2.6(1.4)	22.3(16.9)	35.8(18.5)
30-39	8.15(0.4)	2.6(1.4)	28.8(28.8)	36.7(19.7)
40-49	8.16(0.3)	2.4(1.3)	32.0(31.3)	34.6(17.3)
50-59	8.13(0.3)	2.1(1.1)	36.9 (36.5)	34.9(17.1)
60 and above	8.26(0.3)	1.7(0.9)	34.2(30.2)	36.6(23.3)
p-value	0.47	3.41e-05	0.01	0.53

The table shows the seminal fluid characteristics compared with the age groups of study participants *p-value<0.05

## Discussion

This study aimed to evaluate the seminal fluid profile of male partners of infertile couples who have been offered fertility treatment at a fertility center in Ghana, to identify the types of semen fluid abnormalities. From our study, the most common semen abnormalities were oligospermia, teratozoospermia, asthenozoospermia, hyperviscous semen, and hypospermia. Additionally, the mean (SD) of semen volume and sperm concentration differed significantly between the various age groups. Semen abnormalities may manifest in the volume, viscosity, pH, sperm concentration, motility, and morphology, and each of these abnormalities, based on their severity, has implications for male infertility.

Semen viscosity is known to play a significant role in the fertility ability of the spermatozoa. Abnormal viscosity must not be discounted in male infertility assessment if there are spermatozoa in the ejaculate. Our study showed a high rate of normal semen viscosity of 80.9% and a semen hyperviscosity (SHV) rate of 18.5%. The semen hyperviscosity (SHV) rate of 18.5% agrees with rates of between 12 - 29% reported by other studies [7-9]. Even though these rates may seem low, they must not be discounted in the treatment of male infertility. In the presence of spermatozoa, normal semen viscosity is known to play a critical role in sperm function and the fertilization process. It facilitates the entry of spermatozoa into cervical mucus [10], maintains sperm swimming speed after mucus penetration, regulates the distribution of surface charges on the sperm membrane during the maturation process [11], prevents the lipid peroxidation reaction [12], and maintains the chromatin integrity of spermatozoa [13]. Even though the rate of SHV was relatively low, it has been shown to be a contributor to poor sperm motility and semen quality, as well as having adverse effects on assisted reproductive technology (ART) cycles. It is a cause of poor outcomes with in vitro fertilization cycles. [14,15]. Hypofunction of the prostate gland and seminal vesicles, as well as leukospermia and bacteriospermia, are the major causes of SHV [14]. Therefore, it is recommended that in the presence of SHV, an assessment of white cell count (WBC) and leucocytes in the semen sample is required. Additionally, the semen sample can be cultured for bacterial isolation and antibiotic sensitivity so that the appropriate antimicrobial therapy can be instituted to aid treatment and subsequently reduce the adverse effects of SHV.

Another important parameter that contributes to male fertility is the sperm count. In our study, a greater portion (64%) of our clients had normal sperm count, and the rest had abnormal sperm count reports (30.5% oligospermia and 5.5% azoospermia). Even though the oligo-zoospermia rate in our study agrees with those of studies in Jos, Nigeria by Imade *et al*. in 2000, and in Paris, France by Auger *et al*. in 1995 [16,17], when compared with an earlier study in Nigeria that had reported a rather higher rate of oligospermia/azoospermia of 74% [18], the rate of abnormal sperm count in our cohort was very low. An Indian study, however, reported a high prevalence of azoospermia of 14.28% but a lower rate of oligospermia of 21.43% [19]. We hereby postulate that the low rate of oligospermia and azoospermia in our study population compared to that in the Nigerian study (in the same West African subregion) could imply that infertility may not be mainly associated with problems with male factors but other aetiological factors, such as environmental and female factors, may play a major role and therefore a thorough fertility workup for these couples may reveal the actual cause of the problem.

Several studies have evaluated the effect of advancing male age on semen quality. A Ghanaian study over an eleven-year period (1995 - 2005), showed a downward trend in the sperm concentration of males in the reproductive age range of 24 - 36 years in Tema and the surrounding suburbs [20]. Other studies in Nigeria also reported a progressive decline [21], and they postulated that the decline may be linked with environmental, nutritional, socio-economic, or other factors [22,23]. Several reports from other parts of the world (Spain, Scotland, France, Norway, Italy, Denmark, Belgium, Germany, Austria, Greece, Israel, Tunisia, China, and Canada) found that sperm concentrations of men from these countries have been diminishing over time [24]. Our study, however, did not look at this aspect, and so a prospective study will be embarked upon to assess the effects of advancing male age on semen in our population.

The morphology and motility of spermatozoa are important parameters assessed during semen analysis. According to the World Health Organization (WHO), a morphology of ≥4% is normal. Morphology of the spermatozoa describes the differential development of the head, midpiece, and tail. This is a function of the testes as well as the epididymis. Abnormal sperm motility implies that the sperm will be unable to ascend the female genital tract for fertilization. Sperm motility is, therefore, a marker of sperm quality and an indicator of the fertility potential. The more progressive the motility, the higher the fertility potential. In our study, the most common morphological abnormality was teratozoospermia (28.8%), whilst the most common motility abnormality observed in this study was asthenozoospermia (27.3%). Out of our cohort, 63.8% had sperm with normal forward progressive motility. This is similar to a study that reported asthenozoospermia as the most common semen abnormality [25].

The study thus determined that oligozoospermia and asthenozoospermia might be the two factors causing male infertility in the study area, and this agrees with the findings of Imade *et al*. (2000) in Nigeria [16]. This study also shows that a greater percentage, 56.7% (more than half) of the study population, were very young (less than 40 years), and 88.0% were < 50 years old. This implies that younger males are seeking fertility treatment with their partners. There is a need to study the causes of abnormal semen parameters in these younger men.

This study has obvious limitations. This is a retrospective study and suffers from the limitation of missing data, which might affect the results. Furthermore, this is a facility-based study and may not reflect what happens in the general population. Additionally, the laboratory data did not include information on the occupation, medical, and surgical history of the clients. Such information would have been useful in drawing a causal link between these factors and the fertility status of the clients. However, this study serves as a prelude to a prospective study into male infertility in our center.

## Conclusion

This study, to identify the types of semen fluid abnormalities in our male partners of couples who had semen analysis in our center, showed that the rate of abnormal semen quality was 36%. Male factor is still a common cause of infertility among infertile couples seeking treatment, and the most common semen abnormalities were oligospermia, teratozoospermia, asthenozoospermia, hyperviscous semen, and hypospermia. Oligozoospermia and asthenozoospermia might be the two factors causing male infertility in our environment. More young men are seeking fertility treatment with their partners. Therefore, it is imperative to encourage young male partners not to shy away when it comes to the evaluation of infertility and to focus on educational programs targeted at the prevention and management of male infertility.

### What is known about this topic

- Semen analysis is a simple, inexpensive, and reliable means of assessing male factor infertility;
- Semen abnormalities can affect sperm count, sperm motility, and sperm morphology.

### What this study adds

- Categorizes for the first time the type of semen abnormalities of patients assessed for fertility care at a fertility center in Kumasi, Ghana;
- Younger males are seeking fertility treatment with their partners;
- Proposes the need not to ignore young males when it comes to education on semen abnormalities.
